# The role of stress hyperglycemia in in-hospital new-onset atrial fibrillation among patients with acute myocardial infarction: a narrative review

**DOI:** 10.3389/fcvm.2026.1748943

**Published:** 2026-01-27

**Authors:** Xin Wei, Yanxiang Sun

**Affiliations:** 1 Department of Cardiovascular Medicine, Zhongshan People’s Hospital, Zhongshan, China; 2 Shenzhen University Medical School, Shenzhen University, Shenzhen, China

**Keywords:** acute myocardial infarction, major adverse cardiovascular events, new-onset atrial fibrillation, stress hyperglycemia, stress hyperglycemia ratio

## Abstract

Stress hyperglycemia (SHG) is frequently observed in patients with acute myocardial infarction (AMI). Substantial evidence has established both SHG and the stress hyperglycemia ratio (SHR) as significant, independent predictors of adverse outcomes, linking them to an increased risk of major adverse cardiovascular events and demonstrating a strong association with in-hospital new-onset atrial fibrillation (NOAF). This review consolidates epidemiological evidence linking SHG to these clinical endpoints and details the key underlying pathophysiological mechanisms by which SHG promotes NOAF, including inflammatory activation, oxidative stress activation, calcium handling dysfunction, and autonomic remodeling. Future research should prioritize standardizing diagnostic criteria for SHG, developing integrated dynamic prediction models that incorporate SHG/SHR for NOAF risk, and conducting targeted clinical trials to evaluate early interventions.

## 1 Introduction

Acute myocardial infarction (AMI), the most severe and life-threatening manifestation of coronary artery disease, imposes a devastating global burden and stands as a foremost cause of mortality worldwide (1, 2). Within this context, elevated admission blood glucose emerges as a remarkably prevalent clinical finding. Epidemiological studies have reported a common prevalence of elevated admission glucose in AMI patients, ranging from 25% to 50% (3–5). This transient elevation of blood glucose secondary to acute stress is termed stress hyperglycemia (SHG) (6). It can manifest both in individuals without a prior diabetes diagnosis and in diabetic patients, even in those whose blood glucose was previously well-controlled (7, 8). As an adaptive survival mechanism, SHG establishes a new glycemic equilibrium to maximize glucose utilization under stress, thus partially mitigating ischemic injury and, as a transient mechanism, typically resolving after the acute stressor subsides (9). Accumulating evidence further indicates that SHG and the stress hyperglycemia ratio (SHR) serve not only as predictors of major adverse cardiovascular events (MACE), but are also strongly associated with the occurrence of in-hospital new-onset atrial fibrillation (NOAF) (10–12). Accordingly, this narrative review systematically summarizes the evidence and recent advances regarding the relationship between SHG and these clinical endpoints, with a particular focus on NOAF in the setting of AMI, and explores the underlying pathophysiological mechanisms to provide insights for future research. While the primary focus is on AMI-associated in-hospital NOAF, the mechanistic insights discussed may also inform the understanding of hyperglycemia-related arrhythmogenesis in other acute cardiovascular conditions.

## 2 Association between SHG and cardiovascular outcomes in AMI patients

Despite its high clinical prevalence and prognostic significance in the acute phase of AMI, SHG remains poorly recognized and managed due to the absence of universally accepted diagnostic criteria. Consequently, in previous research, biomarkers such as admission glucose, fasting plasma glucose (FPG), and glycated hemoglobin A1c (HbA1c) were commonly employed as quantitative measures of acute dysglycemia in AMI patients (4, 13–18). Critically, SHG is robustly associated with elevated all-cause mortality, both short- and long-term after an AMI (4, 12). In the following sections, we will review and synthesize the evidence connecting SHG with the risks of MACE and NOAF.

### 2.1 Association between SHG and MACE

MACE is a composite endpoint that encompasses cardiogenic shock, cardiac arrest, recurrent myocardial infarction, malignant arrhythmias, heart failure, and stroke (19–21). Studies on the association between glycemic levels and MACE were summarized (Table 1). Horst et al. firstly linked post-AMI hyperglycemia to MACE in 2007 (13). Their investigation, which followed 417 percutaneous coronary intervention (PCI)-treated AMI patients for 30 days, demonstrated that persistent in-hospital hyperglycemia significantly correlated with a higher MACE risk (HR 1.12, 95% CI 1.04–1.20) in the overall cohort, pointing to its potential as an independent predictor for short-term adverse events. Subsequently, Kitada et al. identified 2 hour post-load plasma glucose ≥160 mg/dL (OR 1.85, 95% CI 1.07–3.21) as an independent predictor of subsequent MACE risk within 2 years in AMI patients (14). Furthermore, Eitel et al. conducted a prospective analysis of ST-segment elevation myocardial infarction (STEMI) patients who received primary PCI and identified a significant association between increased admission glucose and escalated long-term MACE risk (HR 2.6, 95% CI 1.6–4.4, *P* < 0.001) (16), and the result was validated in other populations (4, 17, 18). Beyond static measures, glycemic variability (GV), quantified via metrics such as the mean amplitude of glycemic excursions and standard deviation (SD) derived from continuous glucose monitoring, has emerged as an independent prognostic predictor. Tokue et al. revealed that glycemic fluctuation patterns within 48 h post-PCI in STEMI patients were strongly associated with 180-day major adverse cardiac and cerebrovascular events (MACCE) (22). Compared to a normoglycemic group, intermittent hyperglycemia and persistent hyperglycemia increased MACCE risk by 5.32-fold (HR = 5.32, 95% CI 1.08–26.45) and 7.73-fold (HR = 7.73, 95% CI 1.09–54.77), respectively. Subsequently, both Su et al. and Yang et al. corroborated that high GV during early hospitalization or at admission was a potent, independent predictor of 1-year MACE in AMI patients (Su et al.: HR = 3.107, 95% CI 1.190–8.117, *P* = 0.021; Yang et al.: HR = 3.645, 95% CI 1.287–10.325, *P* = 0.015), whereas HbA1c showed no independent association in either study (15, 23). Additionally, hypoglycemia has also been linked to cardiovascular events. A meta-analysis incorporating 9 RCTs revealed a higher MACE risk (HR = 1.66, 95% CI 1.35–2.06, *P* < 0.01) in patients with hypoglycemia compared to those without, underscoring that avoiding severe hypoglycemia is equally crucial for improving outcomes alongside glycemic control (24). However, none of these conventional and dynamic glycemic indicators is capable of distinguishing the acute hyperglycemic response to stress from chronic dysglycemia due to pre-existing diabetes.

**Table 1:** Published studies on the association between glycemic parameters and MACE.

First author, year	Country	Study group	Follow-up duration	Patients (*n*)	HR/OR	95%CI
Horst et al. 2007 (13)	Netherlands	Primary PCI-Treated AMI	30 days	417	Persistent Hyperglycemia: HR: 1.12 (Overall Cohort) HR: 1.19 (Subgroup without DM) Normoglycemia Cohort (Reference)	CI: 1.04–1.20 (Overall Cohort) CI: 1.05–1.36 (Subgroup without DM)
Kitada et al. 2010 (14)	Japan	AMI Patients with IGT	2 years	422	OR: 1.85	1.07–3.21
Eitel et al. 2012 (16)	Germany	Primary PCI-Treated AMI	19 months^b^	401	HR: 2.6	1.6–4.4
Ekmekci et al. 2014 (17)	Turkey	Primary PCI for STEMI without prior DM	In-Hospital	503	Tertile 3: OR: 9.55^a^ (High Admission Glucose Levels)	1.99–46.5
Stalikas et al. 2022 (18)	Greece	Primary PCI-Treated STEMI	1.7 years^b^	309	HR: 1.802^a^	1.01–3.21
Savic et al. 2024 (4)	Serbia	Primary PCI for STEMI without prior DM	Short-term: 30 days Long-term: 8 years	2,362	Short-term: HR 1.99^a^ Long-term: HR 1.35^a^	Short-term: 1.03–3.85 Long-term: 1.01–1.89
Su et al. 2013 (15)	China	Elderly AMI patients	1 year	186	Tertile 3: HR: 3.107 (High MAGE level)	1.190–8.117
Yang et al. 2024 (23)	China	STEMI patients with DM and HF	1 year	484	HR: 3.645^a^ (High MAGE level)	1.287–10.325
Xu et al. 2022 (7)	China	STEMI patients	30 days	5,417	HR: 1.277^a^ (Per 1-SD increase in SHR)	1.182–1.380
Luo et al. 2024 (8)	China	AMI without Prior AF	2.7 years^b^	2,145	High SHR (≥1.119) + SR: HR: 1.32^a^ Low SHR + SR (Reference)	0.97–1.78
Gao et al. 2023 (26)	China	MINOCA patients	3.5 years^b^	1,179	HR: 2.30^a^ (Per 1-SD increase in SHR)	1.21–4.38

MACE, major adverse cardiovascular events; PCI, percutaneous coronary intervention; AMI, acute myocardial infarction; STEMI, ST-segment elevation myocardial infarction; IGT, impaired glucose tolerance; DM, diabetes mellitus; AF, atrial fibrillation; MINOCA, myocardial infarction with nonobstructive coronary arteries; HR, hazard ratio; OR, odds ratio; SD, standard deviation; MAGE, mean amplitude of glycemic excursions; SHR, stress hyperglycemia ratio; SR, sinus rhythm; CI, confidence interval.

a Adjusted odds ratios/hazard ratios for covariates.

b Median follow-up time.

To overcome the critical limitation, SHR (SHR = admission glucose (mmol/L)/[1.59 × HbA1c (%)−2.59]), developed by Roberts, is a novel composite metric that concurrently reflects acute and chronic glycemic status, providing a more precise characterization of relative SHG and has demonstrated a robust correlation with heightened incidence of MACE in AMI patients (25). Xu et al. demonstrated in a large STEMI population that each increase of SD in SHR significantly elevated 30-day MACE risk (HR 1.277, 95% CI 1.182–1.380, *P* < 0.001). Notably, further subgroup analysis categorized patients by SHR and diabetes mellitus (DM) status, revealing that those with both elevated SHR and pre-existing DM exhibited the highest risk of MACE (HR 1.34, 95% CI 1.130–1.589, *P* = 0.001) (7). Extending beyond these fundamental associations, Luo et al. highlighted that the risk conferred by a high SHR is substantially amplified by the co-occurrence of in-hospital NOAF (HR 1.32, 95% CI 0.97–1.78, *P* < 0.001) (8). Importantly, broadening the etiological scope, Gao et al. specifically evaluated patients with myocardial infarction with nonobstructive coronary arteries and demonstrated that elevated SHR was independently associated with over a two-fold increase in long-term MACE risk (HR 2.30, 95% CI 1.21–4.38, *P* = 0.011), offering superior risk stratification compared to admission glucose alone, particularly in diabetic patients (26).

In summary, the evidence reviewed establishes a spectrum of glycemic derangements, including static hyperglycemia, GV, and relative hyperglycemia quantified by the SHR, as significant predictors of both short- and long-term MACE in AMI patients. Critically, the SHR refines risk stratification by distinguishing acute stress-induced hyperglycemia from chronic dysglycemia, with the highest risk observed in patients exhibiting both an elevated SHR and pre-existing diabetes. Furthermore, the association of hypoglycemia with adverse events underscores the complexity of glycemic management, highlighting the need for strategies that mitigate hyperglycemic risk without inducing harmful glucose lows. However, significant heterogeneity in defining and applying these glycemic metrics persists, and their prognostic utility in high-risk AMI subpopulations (e.g., those with renal or hepatic comorbidities) remains inadequately characterized. Future research should therefore prioritize standardizing these metrics, elucidating their role in vulnerable cohorts, and defining optimal glycemic control protocols that balance efficacy with safety.

### 2.2 Association between SHG and NOAF

NOAF is a common but underestimated complication with an in-hospital incidence ranging from 3.48% to 16.9% (27). Previous studies have highlighted the occurrence of AF is associated with subsequent risk of adverse outcomes, including cardiogenic stroke, heart failure, malignant arrhythmia and mortality (28–31). Although the link between SHG and NOAF remains less explored than that with MACE (Table 2), early evidence points to an increased incidence of NOAF in AMI patients with in-hospital hyperglycemia (31–33). Koracevic et al. demonstrated that AMI patients with admission glucose ≥8.0 mmol/L exhibited significantly higher NOAF incidence vs. normoglycemic counterparts(15% vs. 7.87%, *P* = 0.010). Moreover, the coexistence of hyperglycemia and NOAF was associated with a markedly elevated in-hospital mortality (1.67% vs. 24.14%) (34). Further corroborating this link, Li et al. identified fasting hyperglycemia as a powerful, independent predictor of NOAF through multivariable analysis (OR 2.65, 95% CI 1.53–4.30) (35). Their study additionally demonstrated a graded association, noting a 5% increase in NOAF risk for every 1 mmol/L increment in fasting glucose (OR 1.05, 95% CI 1.00–1.10). To adjust for the confounding effect of diabetes on underlying glycemic status, Pan conducted a large-scale study (*n* = 3,194) and reported a markedly elevated NOAF risk in the highest vs. lowest SHR quartile (OR 1.733, 95% CI 1.130–2.657, *P* = 0.012) (36). Most recently, Luo et al. not only reconfirmed the positive SHR-NOAF association (OR 1.05 per 10% SHR increase, 95% CI 1.01–1.10) but notably demonstrated particularly pronounced effects in non-diabetic patients (OR 1.08, 95% CI 1.01–1.17) (8). Expanding beyond acute hyperglycemia in AMI, evidence suggests that chronic glycemic instability also constitutes a pro-arrhythmic risk factor. A large cohort study of 27,246 patients with type 2 DM (median follow-up 70.7 months) found that higher long-term visit-to-visit GV, measured by HbA1c variability score, was independently associated with an increased risk of NOAF (HR 1.29, 95% CI 1.12–1.50, *P* < 0.001) (37). Collectively, this evidence establishes that dysglycemia, including both acute stress-induced hyperglycemia and chronic glucose fluctuations, is a significant, independent contributor to AF risk, with the SHR serving as a pivotal metric for quantifying the acute risk component specifically in AMI.

**Table 2:** Published studies on the association between glycemic parameters and NOAF.

First author, year	Country	Study group	Follow-up duration (years)	Patients (*n*)	OR	95%CI
Koracevic et al. 2008 (34)	Serbia	AMI without Prior AF	In-hospital	543	admission glucose ≥8.0 mmol/L: 2.07 DM: 2.04	1.180–3.637 1.06–3.93
Li et al. 2021 (35)	China	AMI without Prior AF	11.2^c^	563	High Fasting Hyperglycemia: 2.56^a^ Per 1 mmol/L increase in Fasting Hyperglycemia: 1.05^a^	High Fasting Hyperglycemia: 1.53–4.30 Per 1 mmol/L increase in Fasting Hyperglycemia: 1.00–1.10
Pan et al. 2022 (22)	China	AMI without Prior AF	5.4	3,194	Quartile 4: 1.733^a^ (High SHR)	1.130–2.657
Luo et al. 2024 (8)	China	AMI without Prior AF	2.7^b^	2,145	Per 10% increase in SHR: 1.05	1.01–1.10

NOAF, new-onset atrial fibrillation; AMI, acute myocardial infarction; AF, atrial fibrillation; SHR, stress hyperglycemia ratio; OR, odds ratio; CI, confidence interval.

a Adjusted odds ratios/95% confidence interval.

b Median follow-up time.

c Average Follow-up.

### 2.3 Predictive performance of glycemic metrics for clinical endpoints

Given that SHR is a biomarker of acute glycemic stress with independent predictive value for multiple post-AMI endpoints, its prognostic performance has been explored in comparative studies of various glycemic metrics (11, 38, 39). Cui et al. and Fu et al. demonstrated that fasting SHR had moderate prognostic utility comparable to FPG for predicting in-hospital mortality in AMI patients, irrespective of diabetes status (AUC 0.689–0.702) (40). However, studies specifically evaluating the predictive utility of SHR for MACE remain limited and have yielded suboptimal results. For instance, data from Luo et al. indicated poor discriminatory power of SHR for MACE (AUC 0.52, 95% CI 0.97–1.78; sensitivity 0.22, specificity 0.87) (8). Similarly, Horst et al. reported only modest predictive performance using admission blood glucose for MACE (AUC 0.59, 95% CI 0.52–0.65) (13). The poor performance might be attributed to a key limitation that SHR is a static, ratio-based snapshot in essence, although it refines the concept of hyperglycemia by accounting for chronic status.

Accordingly, it underscores a key advantage of GV: its ability to capture dynamic glucose fluctuations that static metrics miss. While numerous studies establish GV as a predictor for cardiovascular endpoints including MACE and NOAF, dedicated analyses quantifying its model performance metrics remain scarce (37). For instance, studies seldom benchmark GV's discriminative power against established predictors for MACE, and there is a notable paucity of research evaluating the AUC of GV specifically for predicting in-hospital NOAF or its incremental value over clinical scores. One possible reason is that the indicator itself requires longitudinal frequent measurement of patients' blood glucose level, requiring a sufficiently long follow-up time and high degree of patient compliance to ensure accuracy.

To bridge the gap, a promising path lies in integrating diverse indicators into multivariable prediction models, incorporating both glycemic and non-glycemic indicators. For example, adopting SHR into existing tools or multivariate models, such as the Global Registry of Acute Coronary Events risk score and the CHA2DS2-VASc score, might improve the estimation. Consequently, future predictive models for NOAF may achieve superior, multifaceted risk stratification by concurrently evaluating acute glycemic stress, dynamic glycemic instability, and established clinical risk factors (41–43).

## 3 Potential mechanisms underlying SHG-induced NOAF

The development of SHG entails a complex pathophysiological process driven by neuroendocrine activation, inflammatory responses, and insulin resistance (6, 44). This acute dysmetabolism is associated with adverse outcomes in patients with AMI (45–48). However, the precise mechanisms by which SHG induces NOAF remain incompletely elucidated. Current evidence indicates that SHG and GV trigger a pathophysiological cascade encompassing four interrelated core processes: inflammatory activation, oxidative stress activation, calcium handling dysfunction, and autonomic remodeling (49, 50). These mechanisms synergistically promote atrial fibrosis and electrical remodeling, ultimately leading to the development of AF (51–53).

### 3.1 Upregulation of inflammatory cytokines

Systemic inflammation is implicated in the initiation and perpetuation of AF. A robust inflammatory response is an integral part of tissue injury during AMI. Multiple studies have established that acute elevations in inflammatory markers (e.g., C-reactive protein, interleukins) are strongly associated with an increased risk of NOAF in these patients, both during hospitalization and at one-year follow-up (54, 55). Furthermore, SHG serves as a key driver of this inflammatory activation during the acute phase. It can significantly raise plasma levels of key cytokines, including interleukin-6 (IL-6), tumor necrosis factor-α (TNF-α), and interleukin-18 (IL-18), within two hours with levels promptly decline upon restoration of normoglycemia (54, 56–59). Notably, this hyperglycemia-induced inflammatory response can be suppressed by antioxidants such as glutathione, suggesting that oxidative stress acts as an upstream trigger (58).

**Figure 1:**
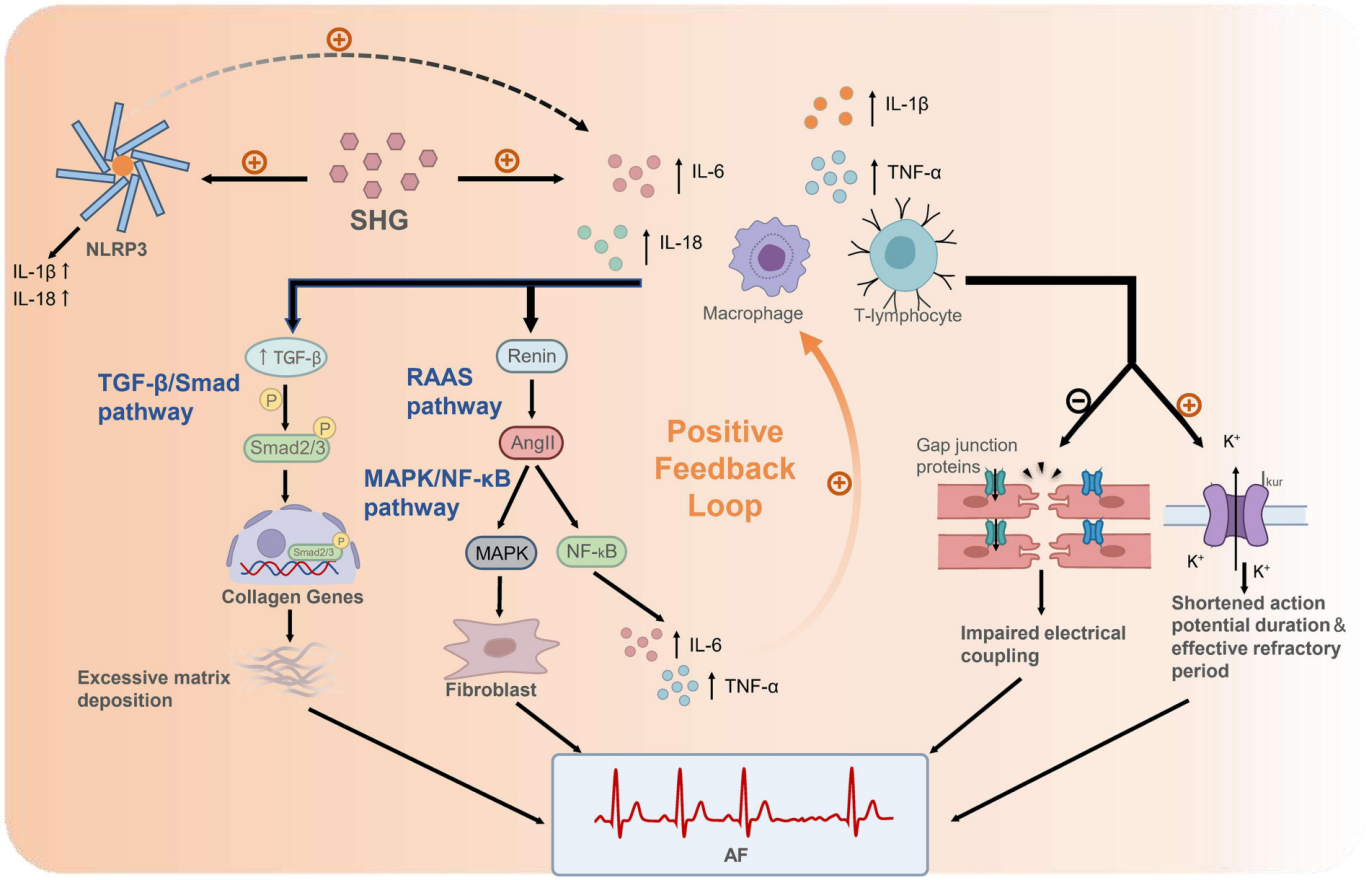
Mechanisms linking SHG-induced inflammation to AF following AMI. SHG rapidly elevates pro-inflammatory cytokines (e.g., IL-6, TNF-α, IL-1β/IL-18). These cytokines promote fibrosis primarily by activating the TGF-β/Smad pathway, wherein TGF-β induces Smad2/3 phosphorylation. The resulting Smad complex translocates into the nucleus and initiates transcription of collagen genes, leading to excessive extracellular matrix deposition. Inflammatory cytokines also stimulate local renin release, activating the RAAS pathway. The resultant Ang II activates the MAPK/NF-κB pathway, which promotes fibroblast activation through MAPK kinases and induces NF-κB signaling. NF-κB drives transcription of cytokines such as IL-6 and TNF-α, establishing a positive feedback loop that further amplifies inflammation. In parallel, inflammatory mediators induce electrical remodeling by downregulating gap junction proteins, thereby impairing cell-cell coupling, and by upregulating potassium currents (e.g., I_kur_), which shortens the atrial action potential duration and effective refractory period. Furthermore, SHG activates the NLRP3 inflammasome. The activated NLRP3 inflammasome directly releases pro-inflammatory cytokines such as IL-1β and IL-18 and amplifies the expression of other inflammatory mediators including IL-6 and TNF-α. These interconnected pathways cooperatively drive structural and electrical remodeling of the atria, ultimately culminating in AF. SHG, stress hyperglycemia; IL-6, interleukin-6; TNF-α, tumor necrosis factor-alpha; IL-1β, interleukin-1 β; TGF-β, transforming growth factor-β; RAAS, renin-angiotensin-aldosterone system; Ang II, angiotensin II; MAPK, mitogen-activated protein kinase; NF-κB, nuclear factor kappa-light-chain-enhancer of activated B cells; I_kur_, ultra-rapid delayed rectifier potassium current; NLRP3, NOD-like receptor family, pyrin domain containing 3; AF, atrial fibrillation.

Figure 1 illustrates the key pathways through which SHG, by activating an inflammatory response, leads to the occurrence of AF. Elevated inflammatory cytokines collectively establish a pathological microenvironment conducive to AF initiation and maintenance through several interconnected pathways (53, 60). The mechanisms involve driving structural remodeling, inducing electrical alterations, and engaging in crosstalk with other pathways. Atrial structural remodeling is driven by inflammatory cells (e.g., macrophages, T lymphocytes) and cytokines (e.g., IL-1β, TNF-α) through an interconnected signaling network (54). In particular, the TGF-β/Smad pathway plays a central role in fibrosis. Inflammatory cells release cytokines that activate this pathway, thereby promoting collagen deposition and fibrotic remodeling. These cytokines also stimulate the local renin-angiotensin-aldosterone system (RAAS), leading to angiotensin II (Ang II)-mediated activation of the MAPK/NF-κB pathway. This, in turn, further amplifies the production of cytokines such as IL-6 and TNF-α, enhances fibroblast activity, and establishes a positive feedback loop. In parallel, electrical remodeling is induced as inflammatory mediators directly or indirectly alter the electrophysiological properties of cardiomyocytes. For instance, cytokines such as TNF-α can downregulate or disrupt the function of gap junction proteins (e.g., connexin 40/43), thereby impairing electrical coupling between cells. Concurrently, the inflammatory response can upregulate the function of channels such as the ultra-rapid delayed rectifier potassium current (I_k__u__r_), leading to a shortening of the atrial action potential duration and effective refractory period. These changes not only facilitate the formation of reentrant circuits but may also synergize with calcium handling abnormalities to increase the propensity for triggered activity (59, 61, 62). Critically, this inflammatory upregulation establishes a vicious cycle with other mechanisms. It does not occur in isolation. Recent mechanistic studies indicate that the inflammatory state triggered by SHG is closely associated with the activation of the NOD-like receptor family, pyrin domain containing 3 (NLRP3) inflammasome (63, 64). The activated NLRP3 inflammasome not only directly releases pro-inflammatory cytokines such as IL-1β and IL-18 but also amplifies the expression of other inflammatory mediators including IL-6 and TNF-α. Furthermore, the inflammatory response extensively interacts with other pathological processes, such as oxidative stress, Calmodulin-dependent protein kinase II (CaMKII) pathway activation, and autonomic remodeling. These interconnected mechanisms form a mutually reinforcing network that continuously drives both structural and electrical remodeling of the atria, ultimately facilitating the initiation and perpetuation of atrial fibrillation (60, 65).

### 3.2 Activation of oxidative stress

Hyperglycemia induces excessive production of reactive oxygen species (ROS) (66). When ROS production surpasses the scavenging capacity of endogenous antioxidant enzymes, including superoxide dismutase and glutathione peroxidase, the redox homeostasis is disrupted, resulting in widespread cellular damage. This pathological process is termed oxidative stress (66, 67). Oxidative stress can cause mitochondrial dysfunction and ion channel abnormalities, thereby increasing susceptibility to abnormal electrical activity in the atria (53, 68). Animal studies provide direct evidence for these mechanisms. In diabetic model rats, compared with the glucose-controlled group, the uncontrolled glucose group exhibited more pronounced cardiac fibrosis (evidenced by increased expression of collagen type 1, collagen type 3, and α-smooth muscle actin) and a higher inducibility of atrial fibrillation. Glycemic fluctuation further exacerbated these pathological changes (69). Similarly, in a mouse model of myocardial ischemia/reperfusion, high-glucose perfusion aggravated myocardial injury and enhanced cardiac oxidative stress (63). A key molecular link in these pathological effects is the upregulation of thioredoxin-interacting protein (Txnip) by hyperglycemia or glucose fluctuations. Elevated Txnip promotes ROS generation and cardiomyocyte apoptosis, ultimately driving fibrosis (63, 64, 69, 70).

Critically, as a core pathological nexus, oxidative stress not only directly drives atrial fibrosis but also mediates electrical remodeling and calcium dyshomeostasis by activating a detrimental ROS–ox-CaMKII circuit. Furthermore, it contributes to autonomic nervous system dysfunction and participates in the pathological remodeling of epicardial adipose tissue. These mechanisms cooperatively establish a complex substrate that favors the initiation and maintenance of AF (discussed in Sections 2.3 and 2.4) (71–74).

### 3.3 CaMKII activation and disruption of calcium homeostasis

CaMKII is an enzyme that plays an important regulatory role in the heart and brain. Its chronic activation has been documented in various pathological conditions, including diabetes and heart failure (75). Upregulation of Upregulation of CaMKII promotes cardiac remodeling and elevates the risk of arrhythmias. Moreover, CaMKII serves as a critical node linking metabolic stress to both atrial and ventricular arrhythmogenesis.

**Figure 2:**
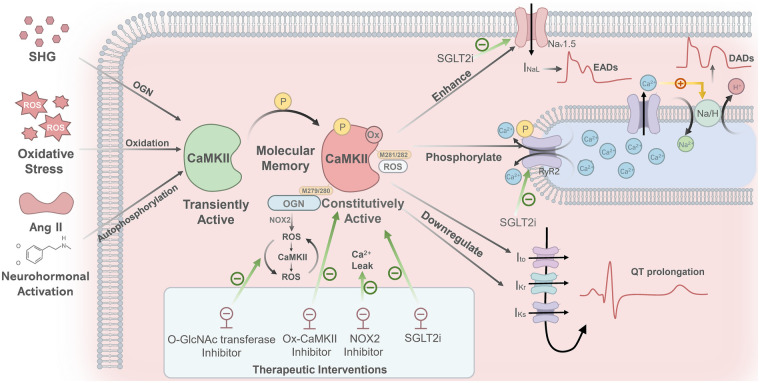
Mechanisms linking CaMKII activation to NOAF. SHG, oxidative stress, and neurohormonal activation (e.g., by angiotensin II and catecholamines) promote persistent CaMKII activation through distinct post-translational modifications: OGN, oxidation, and autophosphorylation, respectively. This shifts CaMKII from a transiently active state into a constitutively active one, establishing a form of “molecular memory.” Specifically, SHG induces OGN at M279/280 of the CaMKII*δ* isoform, which triggers a NOX2-dependent ROS burst and initiates a self-amplifying “ROS–CaMKII–ROS” cycle. Oxidative stress generates ROS that oxidize CaMKII at M281/282, shifting it from a transiently active state into a constitutively active one. The oxidized CaMKII phosphorylates RyR2, leading to increased sarcoplasmic reticulum Ca²^+^ release, which activates the Na/H exchanger and thereby generates DADs. Furthermore, abnormally activated CaMKII enhances the I_NaL_, which prolongs action potential duration and induces EADs. Additionally, CaMKII downregulates several potassium currents (I_to_, I_Kr_, I_Ks_), leading to QT interval prolongation. Inhibitors of O-GlcNAc transferase, ox-CaMKII, or NOX2 can effectively block ROS bursts, suppress oxidized CaMKII, and reduce calcium leakage, respectively. Empagliflozin lowers CaMKII activity and reduces abnormal phosphorylation of both RyR2 and Na_V_1.5. SHG, stress hyperglycemia; CaMKII, calmodulin-dependent protein kinase II; OGN, O-linked β-N-acetylglucosaminylation; ROS, reactive oxygen species; NOX2, NADPH oxidase 2; RyR2, ryanodine receptor 2; DADs, delayed afterdepolarizations; EADs, early afterdepolarizations; I_NaL_, late sodium current; I_to_, transient outward potassium current; I_Kr_, rapid delayed rectifier potassium current; I_Ks_, slow delayed rectifier potassium current; QT, QT Interval; Na_V_1.5, voltage-gated sodium channel 1.5.

Figure 2 illustrates the key pathways whereby SHG and other stimuli trigger AF by activating CaMKII. Under acute pathological conditions, multiple pathological stimuli, such as SHG, oxidative stress, and neurohormonal activation (e.g., by Ang II and catecholamines), can induce persistent and autonomous CaMKII activation. These factors promote persistent CaMKII activation via various post-translational modifications, including O-GlcNAcylation (OGN), oxidation, and autophosphorylation, among others. Consequently, CaMKII shifts from a transiently activated physiological modulator into a constitutively active pathological driver, a transition that establishes a form of “molecular memory”. Aberrantly activated CaMKII promotes arrhythmogenesis through two synergistic pathways. The first pathway provides immediate triggers for abnormal electrical activity: by enhancing the late sodium current (I_NaL_), CaMKII prolongs the action potential, thereby inducing early afterdepolarizations (EADs). Concurrently, through phosphorylation of the ryanodine receptor 2 (RyR2), it increases spontaneous calcium release from the sarcoplasmic reticulum (SR). This elevated cytosolic calcium activates the Na+/H+ exchanger, generating a transient inward current that underlies delayed afterdepolarizations (DADs) (59, 60). The second pathway establishes a sustained pro-arrhythmic substrate: CaMKII downregulates several potassium currents (I_to_, I_Kr_, I_Ks_), thereby reducing the repolarization reserve and prolonging the action potential (manifested as QT prolongation). Furthermore, it promotes inflammation, fibrosis, apoptosis, and mitochondrial dysfunction, collectively impairing cell-to-cell coupling and slowing cardiac conduction (76–78).

A well-established consensus recognizes the pathway “ROS → ox-CaMKII (M281/282) → RyR2 phosphorylation → SR Ca²^+^ leak → DADs → arrhythmia” as a common mechanism contributing to both atrial and ventricular arrhythmias (76–78). More recently, studies in cellular models of acute hyperglycemia have further shown that OGN directly modifies the M279/280 residue of the CaMKII*δ* isoform, inducing its sustained activation. Then it triggers an arrhythmogenic cascade that includes NOX2-dependent ROS burst and RyR2-mediated SR Ca²^+^ leak (71), as well as a “ROS–CaMKII–ROS” vicious cycle (72). In contrast, OGN-modified CaMKII is not detected in chronic diabetes-related atrial fibrillation models. Although OGN is markedly elevated in the diabetic heart, its pro-arrhythmic effect in the atria appears to operate primarily through a pathway independent of and parallel to CaMKII “hyperglycemia → increased OGN → targets other than CaMKII (potentially including RyR2, transcription factors, or structural proteins) → arrhythmia” (78). Thus, the direct modification of CaMKII by OGN may play distinct roles in arrhythmogenesis under acute vs. chronic conditions, as well as between atrial and ventricular tissues. In conclusion, these mechanisms collectively contribute to cardiac structural remodeling and arrhythmogenesis.

Building on these mechanisms, targeting CaMKII and its upstream modification pathways has emerged as a promising therapeutic strategy, with studies providing multi-layered support for precise interventions. On one hand, targeted intervention at key pathological steps in different models shows considerable potential (79, 80). In acute hyperglycemic models, inhibiting O-GlcNAc transferase or NOX2 activity effectively blocks ROS bursts and calcium leakage (71). In chronic diabetes-related atrial fibrillation models, specifically inhibiting ox-CaMKII or enhancing O-GlcNAcase activity to reduce global protein OGN also significantly attenuates arrhythmia (78). On the other hand, some cardiovascular drugs already in clinical use can improve cardiomyocyte electrophysiological and mechanical function. For example, empagliflozin reduces CaMKII activity and the abnormal phosphorylation of its downstream targets (including RyR2 at S2814 and the sodium channel Na_V_1.5 at S571), thereby enhancing calcium handling and electrical stability in cardiomyocytes (81, 82). Moreover, a recent study demonstrates that semaglutide directly suppresses pathological late sodium current and reduces diastolic SR calcium leak, thereby improving myocardial contractility (83).

### 3.4 Autonomic remodeling and epicardial adipose tissue remodeling

Hyperglycemia serves as a key driver of pathological cardiac autonomic remodeling. Under stress, neurotrophic factors released from the myocardium trigger sympathetic nerve sprouting and hyperinnervation in the atria, thereby forming a structural “neural substrate” conducive to AF. Subsequently, the intrinsic cardiac ganglia become hyperactive and act as aberrant integration centers. These ganglia not only receive and process abnormal sympathetic and vagal inputs but also generate spontaneous or simultaneous sympathetic–vagal co-discharges, representing critical initiating events for AF. Specifically, sympathetic overactivation primarily acts through the *β*-adrenergic receptor–Gα_s_–cAMP–PKA/CaMKII pathway. This cascade enhances the L-type calcium current (I_CaL_) and RyR2 activity, while suppressing the inward rectifier potassium current (I_K1_), leading to SR calcium leak and the eventual induction of DADs. In parallel, vagal activation releases acetylcholine, which activates acetylcholine-activated potassium currents. This markedly shortens the atrial effective refractory period. The resulting spatial heterogeneity in refractoriness facilitates the formation of a re-entry-prone substrate. Although functionally antagonistic, the sympathetic and vagal systems can act synergistically. The coexistence of sympathetic-mediated prolongation of calcium transients and vagal-induced shortening of the action potential duration particularly in regions such as the pulmonary veins, readily facilitates the generation of “late phase-3 early afterdepolarizations”, which serve as critical triggering events for AF (73).

Furthermore, remodeling of epicardial adipose tissue (EAT) in metabolic disorders such as obesity and diabetes represents another key mechanism underlying increased AF susceptibility. Through paracrine signaling, EAT releases bioactive substances into the adjacent atrial myocardium, including inflammatory mediators (e.g., TNF-α, IL-6), pro-fibrotic exosomes, and ROS. These factors act in concert to drive both structural remodeling (e.g., fibrosis) and electrical remodeling (e.g., conduction disturbances and increased electrophysiological heterogeneity) of the atrial substrate, thereby creating and sustaining a pathological microenvironment conducive to the initiation and perpetuation of AF (59, 74, 84, 85).

## 4 Therapeutic strategies for AMI with concomitant SHG

The optimal management of SHG in AMI remains a clinically evolving challenge. The core of current intervention strategies lies in balancing the cardiovascular benefits of glycemic control against potential risks such as hypoglycemia. Insulin rapidly corrects hyperglycemia, ameliorates metabolic disturbances, and may attenuate inflammatory responses (86, 87). Early studies, such as the DIGAMI trial, demonstrated that insulin-based therapy reduced mortality in AMI patients, regardless of diabetes status (46, 88–90). However, a subsequent large-scale meta-analysis demonstrated that intensive insulin therapy failed to significantly reduce all-cause mortality (RR 1.03, 95% CI 0.96–1.10) and was associated with an increased risk of iatrogenic hypoglycemia. Notably, iatrogenic hypoglycemia is a recognized independent risk factor for poor prognosis (88). This evidence has tempered the initial enthusiasm for universal intensive insulin therapy, shifting the current clinical focus toward “safe and stable glycemic control,” which aims to maintain reasonable glycemic targets while strictly avoiding hypoglycemia. The advent of novel glucose-lowering agents has reshaped the therapeutic landscape. Drugs such as GLP-1RA, dipeptidyl peptidase-4 inhibitors, and SGLT2i have demonstrated clear benefits in reducing the risk of heart failure hospitalization and MACE in patients with type 2 diabetes or established cardiovascular disease (91–98). In addition to providing cardiovascular protection, these agents carry a substantially lower risk of hypoglycemia compared to insulin, better aligning with the modern therapeutic principle of “benefit-risk balance.” Although preclinical studies suggest that some of these drugs may confer potential atrial protective effects through mechanisms such as mitigating inflammation, suppressing oxidative stress, and improving myocardial electrophysiological stability (68, 81–83, 99), large cardiovascular outcome trials (CVOTs) have not consistently demonstrated a significant reduction in the incidence of atrial fibrillation (100).

In summary, although novel glucose-lowering agents are mechanistically plausible for preventing SHG-associated NOAF, this remains a hypothesis awaiting validation. Future studies need to prospectively pre-specify NOAF as a clinical endpoint in dedicated trials to determine whether targeted interventions can effectively mitigate this risk.

## 5 Conclusion

In summary, this review demonstrates that SHG and the SHR are independent predictors of both MACE and in-hospital NOAF following AMI, primarily through pathways involving inflammatory activation, oxidative stress, calcium handling dysfunction, and autonomic remodeling. Nevertheless, clinical translation remains constrained by the absence of a uniform SHG definition, limited discriminative ability of existing glycemic indices for NOAF, and a shortage of prospective interventional evidence. Consequently, future research must focus on standardizing diagnostic approaches, constructing integrated dynamic prognostic tools, and most critically, implementing targeted trials to evaluate whether pathophysiology-informed strategies can effectively lower NOAF incidence and enhance outcomes in this high-risk population.

### 5.1 Literature search strategy

A systematic literature search was conducted in PubMed, Web of Science, and Embase for studies published up to November 2025. The search strategy combined terms related to acute myocardial infarction (including STEMI and NSTEMI), stress hyperglycemia and the stress hyperglycemia ratio (SHR), as well as in-hospital new-onset atrial fibrillation and major adverse cardiovascular events, using Boolean logic with syntax adapted to each database. As a narrative review, study selection was guided by their relevance to the pathophysiological mechanisms and clinical implications central to this discussion.

## References

[1] Dai H Much AA Maor E Asher E Younis A Xu Y Global, regional, and national burden of ischaemic heart disease and its attributable risk factors, 1990–2017: results from the global burden of disease study 2017. Eur Heart J Qual Care Clin Outcomes. (2022) 8:50–60. 10.1093/ehjqcco/qcaa076 33017008 PMC8728029

[2] Rao SV O'Donoghue ML Ruel M Rab T Tamis-Holland JE Alexander JH 2025 ACC/AHA/ACEP/NAEMSP/SCAI guideline for the management of patients with acute coronary syndromes: a report of the American College of Cardiology/American Heart Association joint committee on clinical practice guidelines. Circulation. (2025) 151:e771–862. 10.1161/CIR.0000000000001328 40014670

[3] Liu J Zhou Y Huang H Liu R Kang Y Zhu T Impact of stress hyperglycemia ratio on mortality in patients with critical acute myocardial infarction: insight from American MIMIC-IV and the Chinese CIN-II study. Cardiovasc Diabetol. (2023) 22:281. 10.1186/s12933-023-02012-1 37865764 PMC10589959

[4] Savic L Mrdovic I Asanin M Stankovic S Lasica R Krljanac G Long-term prognostic impact of stress hyperglycemia in non-diabetic patients treated with successful primary percutaneous coronary intervention. J Pers Med. (2024) 14:591. 10.3390/jpm14060591 38929812 PMC11204510

[5] Alawaji R Musslem M Alshalahi E Alanzan A Sufyani A Alhati M A systematic review and meta-analysis of the effect of hyperglycemia on admission for acute myocardial infarction in diabetic and non-diabetic patients. Diabetol Metab Syndr. (2024) 16:224. 10.1186/s13098-024-01459-w 39267155 PMC11391676

[6] Song G Liu X Lu Z Guan J Chen X Li Y Relationship between stress hyperglycaemic ratio (SHR) and critical illness: a systematic review. Cardiovasc Diabetol. (2025) 24:188. 10.1186/s12933-025-02751-3 40317019 PMC12049067

[7] Xu W Yang YM Zhu J Wu S Wang J Zhang H Predictive value of the stress hyperglycemia ratio in patients with acute ST-segment elevation myocardial infarction: insights from a multi-center observational study. Cardiovasc Diabetol. (2022) 21:48. 10.1186/s12933-022-01479-8 35351149 PMC8962934

[8] Luo J Li Z Qin X Zhang X Liu X Zhang W Association of stress hyperglycemia ratio with in-hospital new-onset atrial fibrillation and long-term outcomes in patients with acute myocardial infarction. Diabetes Metab Res Rev. (2024) 40:e3726. 10.1002/dmrr.3726 37712510

[9] Srinivasan V. Glucose metabolism and stress hyperglycemia in critically ill children. Indian J Pediatr. (2023) 90:272–9. 10.1007/s12098-022-04439-y 36645581

[10] Meng W Qiu H Li W Li H. Correlation between stress hyperglycemia ratio and prognosis in acute myocardial infarction patients following percutaneous coronary intervention. Front Cardiovasc Med. (2025) 12:1493635. 10.3389/fcvm.2025.1493635 40416812 PMC12098508

[11] Yan N Wu P Zhang Z Wang M Ma J Ma A The association between stress hyperglycemia ratio and 1-year outcomes in patients with acute myocardial infarction: a retrospective large sample cohort study. Front Endocrinol (Lausanne). (2025) 16:1586541. 10.3389/fendo.2025.1586541 40303637 PMC12037398

[12] Alkatiri AH Qalby N Mappangara I Zainal ATF Cramer MJ Doevendans PA Stress hyperglycemia and poor outcomes in patients with ST-elevation myocardial infarction: a systematic review and meta-analysis. Front Cardiovasc Med. (2024) 11:1303685. 10.3389/fcvm.2024.1303685 38529334 PMC10961461

[13] van der Horst IC Nijsten MW Vogelzang M Zijlstra F. Persistent hyperglycemia is an independent predictor of outcome in acute myocardial infarction. Cardiovasc Diabetol. (2007) 6:2. 10.1186/1475-2840-6-2 17284309 PMC1802732

[14] Kitada S Otsuka Y Kokubu N Kasahara Y Kataoka Y Noguchi T Post-load hyperglycemia as an important predictor of long-term adverse cardiac events after acute myocardial infarction: a scientific study. Cardiovasc Diabetol. (2010) 9:75. 10.1186/1475-2840-9-75 21070650 PMC2996353

[15] Su G Mi SH Li Z Tao H Yang HX Zheng H. Prognostic value of early in-hospital glycemic excursion in elderly patients with acute myocardial infarction. Cardiovasc Diabetol. (2013) 12:33. 10.1186/1475-2840-12-33 23399749 PMC3608222

[16] Eitel I Hintze S de Waha S Fuernau G Lurz P Desch S Prognostic impact of hyperglycemia in nondiabetic and diabetic patients with ST-elevation myocardial infarction: insights from contrast-enhanced magnetic resonance imaging. Circ Cardiovasc Imaging. (2012) 5:708–18. 10.1161/CIRCIMAGING.112.974998 23051889

[17] Ekmekci A Cicek G Uluganyan M Gungor B Osman F Ozcan KS Admission hyperglycemia predicts inhospital mortality and major adverse cardiac events after primary percutaneous coronary intervention in patients without diabetes mellitus. Angiology. (2014) 65:154–9. 10.1177/0003319713488930 23657174

[18] Stalikas N Papazoglou AS Karagiannidis E Panteris E Moysidis D Daios S Association of stress induced hyperglycemia with angiographic findings and clinical outcomes in patients with ST-elevation myocardial infarction. Cardiovasc Diabetol. (2022) 21:140. 10.1186/s12933-022-01578-6 35883091 PMC9327277

[19] Kerola AM Juonala M Kytö V. Cardiovascular outcomes of patients with type 2 diabetes after myocardial infarction and the impact of diabetes duration: a nationwide registry study. Diabetes Res Clin Pract. (2025) 228:112411. 10.1016/j.diabres.2025.112411 40782841

[20] Yu Q Wang S Zhao Y Zhao Y Zeng J Wang Y Impact of acute total occlusion of the culprit vessel on prognosis and risk stratification in patients with non-ST-segment elevation myocardial infarction. Am J Cardiol. (2025) 254:139–46. 10.1016/j.amjcard.2025.07.042 40782984

[21] Bucci T Lam SHM Argyris AA Beevers DG Shantsila E Shantsila A Long-term risk of adverse events in patients discharged alive after hospitalization for hypertensive crisis. J Hypertens. (2025) 43:1703–10. 10.1097/HJH.0000000000004113 40778679 PMC12404630

[22] Tokue M Iijima R Imamura T Niikura H Hayashi F Yazaki Y Impact of glycemic variability in patients with ST-elevated myocardial infarction. Int J Cardiol. (2015) 187:660–2. 10.1016/j.ijcard.2015.03.365 25880405

[23] Yang X Su G Zhang T Yang H Tao H Du X Comparison of admission glycemic variability and glycosylated hemoglobin in predicting major adverse cardiac events among type 2 diabetes patients with heart failure following acute ST-segment elevation myocardial infarction. J Transl Int Med. (2024) 12:188–96. 10.2478/jtim-2024-0006 38978967 PMC11229884

[24] Malik AH Yandrapalli S Aronow WS Jain D Frishman WH Panza JA Severe hypoglycemia and risk of subsequent cardiovascular events: systematic review and meta-analysis of randomized controlled trials. Cardiol Rev. (2020) 28:244–9. 10.1097/CRD.0000000000000276 31868770

[25] Roberts GW Quinn SJ Valentine N Alhawassi T O'Dea H Stranks SN Relative hyperglycemia, a marker of critical illness: introducing the stress hyperglycemia ratio. J Clin Endocrinol Metab. (2015) 100:4490–7. 10.1210/jc.2015-2660 26485219

[26] Gao S Huang S Lin X Xu L Yu M. Prognostic implications of stress hyperglycemia ratio in patients with myocardial infarction with nonobstructive coronary arteries. Ann Med. (2023) 55:990–9. 10.1080/07853890.2023.2186479 36896774 PMC10795641

[27] Demidova MM Baturova MA Erlinge D Platonov PG. New-onset atrial fibrillation during ST-segment-elevation myocardial infarction: risk of recurrence and its clinical impact during 10 years of follow-up. J Am Heart Assoc. (2025) 14:e040478. 10.1161/JAHA.124.040478 40970535 PMC12684506

[28] Elshaer F Alsaeed AH Alfehaid SN Alshahrani AS Alduhayyim AH Alsaleh AM. Incidence, clinical predictors, and clinical effect of new-onset atrial fibrillation in myocardial infarction patients: a retrospective cohort study. Saudi Med J. (2022) 43:933–40. 10.15537/smj.2022.43.8.20220349 35964949 PMC9749668

[29] Parashar S Kella D Reid KJ Spertus JA Tang F Langberg J New-onset atrial fibrillation after acute myocardial infarction and its relation to admission biomarkers (from the TRIUMPH registry). Am J Cardiol. (2013) 112:1390–5. 10.1016/j.amjcard.2013.07.006 24135301 PMC4323174

[30] Batra G Svennblad B Held C Jernberg T Johanson P Wallentin L All types of atrial fibrillation in the setting of myocardial infarction are associated with impaired outcome. Heart. (2016) 102:926–33. 10.1136/heartjnl-2015-308678 26928408

[31] Kadri Z Danchin N Vaur L Cottin Y Gueret P Zeller M Major impact of admission glycaemia on 30 day and one year mortality in non-diabetic patients admitted for myocardial infarction: results from the nationwide French USIC 2000 study. Heart. (2006) 92:910–5. 10.1136/hrt.2005.073791 16339808 PMC1860714

[32] Dziewierz A Giszterowicz D Siudak Z Rakowski T Dubiel JS Dudek D. Admission glucose level and in-hospital outcomes in diabetic and non-diabetic patients with acute myocardial infarction. Clin Res Cardiol. (2010) 99:715–21. 10.1007/s00392-010-0175-1 20458486

[33] Terlecki M Bednarek A Kawecka-Jaszcz K Czarnecka D Bryniarski L. Acute hyperglycaemia and inflammation in patients with ST segment elevation myocardial infarction. Kardiol Pol. (2013) 71:260–7. 10.5603/KP.2013.0038 23575781

[34] Koracevic GP Petrovic S Damjanovic M Stanojlovic T. Association of stress hyperglycemia and atrial fibrillation in myocardial infarction. Wien Klin Wochenschr. (2008) 120:409–13. 10.1007/s00508-008-0983-8 18726666

[35] Li M Gao Y Guo K Wu Z Lao Y Li J Association between fasting hyperglycemia and new-onset atrial fibrillation in patients with acute myocardial infarction and the impact on short- and long-term prognosis. Front Cardiovasc Med. (2021) 8:667527. 10.3389/fcvm.2021.667527 34277729 PMC8280294

[36] Pan L Li Z Li C Dong X Hidru TH Liu F Stress hyperglycemia ratio and neutrophil to lymphocyte ratio are reliable predictors of new-onset atrial fibrillation in patients with acute myocardial infarction. Front Cardiovasc Med. (2022) 9:1051078. 10.3389/fcvm.2022.1051078 36440053 PMC9681791

[37] Hsu JC Yang YY Chuang SL Yu CC Lin LY. Higher long-term visit-to-visit glycemic variability predicts new-onset atrial fibrillation in patients with diabetes mellitus. Cardiovasc Diabetol. (2021) 20:148. 10.1186/s12933-021-01341-3 34301257 PMC8305511

[38] Okita S Saito Y Yaginuma H Asada K Goto H Hashimoto O Impact of the stress hyperglycemia ratio on heart failure and atherosclerotic cardiovascular events after acute myocardial infarction. Circ J. (2025) 89:340–6. 10.1253/circj.CJ-24-0612 39443128

[39] Qiao Z Bian X Song C Zhang R Yuan S Lin Z High stress hyperglycemia ratio predicts adverse clinical outcome in patients with coronary three-vessel disease: a large-scale cohort study. Cardiovasc Diabetol. (2024) 23:190. 10.1186/s12933-024-02286-z 38824608 PMC11144339

[40] Cui K Fu R Yang J Xu H Yin D Song W The impact of fasting stress hyperglycemia ratio, fasting plasma glucose and hemoglobin A1c on in-hospital mortality in patients with and without diabetes: findings from the China acute myocardial infarction registry. Cardiovasc Diabetol. (2023) 22:165. 10.1186/s12933-023-01868-7 37403082 PMC10320917

[41] Xiong S Luo Y Chen Q Chen Y Su H Long Y Adjustment of the GRACE score by the stress hyperglycemia ratio improves the prediction of long-term major adverse cardiac events in patients with acute coronary syndrome undergoing percutaneous coronary intervention: a multicenter retrospective study. Diabetes Res Clin Pract. (2023) 198:110601. 10.1016/j.diabres.2023.110601 36871875

[42] Chen Q Su H Yu X Chen Y Ding X Xiong B The stress hyperglycemia ratio improves the predictive ability of the GRACE score for in-hospital mortality in patients with acute myocardial infarction. Hellenic J Cardiol. (2023) 70:36–45. 10.1016/j.hjc.2022.12.012 36586422

[43] Luo J Xu S Li H Li Z Gong M Qin X Prognostic impact of stress hyperglycemia ratio in acute myocardial infarction patients with and without diabetes mellitus. Nutr Metab Cardiovasc Dis. (2022) 32:2356–66. 10.1016/j.numecd.2022.07.004 35965248

[44] Guan Y Liu G Tang F Wu X Shi J Huang Q. Stress hyperglycemia in acute pancreatitis: from mechanisms to prognostic implications. Life Sci. (2025) 365:123469. 10.1016/j.lfs.2025.123469 39956188

[45] Li M Wang J Ding S Ding B Oketunbi TJ Song X Cardiac magnetic resonance imaging-derived pathophysiology and prognosis of diabetes mellitus with acute myocardial infarction after revascularization: a prospective cohort study. Ann Med. (2024) 56:2399751. 10.1080/07853890.2024.2399751 39253848 PMC11571787

[46] Pepe M Addabbo F Cecere A Tritto R Napoli G Nestola PL Acute hyperglycemia-induced injury in myocardial infarction. Int J Mol Sci. (2024) 25:8504. 10.3390/ijms25158504 39126075 PMC11313474

[47] Bamarinejad A Kermani-Alghoraishi M Soleimani A Roohafza H Yazdekhasti S MirmohammadSadeghi A Long-term outcome and prognostic value of angiographic slow/no-reflow phenomenon after emergency percutaneous coronary intervention for ST-elevation myocardial infarction. Coron Artery Dis. (2024) 35:389–96. 10.1097/MCA.0000000000001362 38563194

[48] Okazaki T Nabeshima T Santanda T Hoshina Y Kondo Y Yaegashi Y Association of relative dysglycemia with hospital mortality in critically ill patients: a retrospective study. Crit Care Med. (2024) 52:1356–66. 10.1097/CCM.0000000000006313 38656278

[49] Goudis CA Korantzopoulos P Ntalas IV Kallergis EM Liu T Ketikoglou DG. Diabetes mellitus and atrial fibrillation: pathophysiological mechanisms and potential upstream therapies. Int J Cardiol. (2015) 184:617–22. 10.1016/j.ijcard.2015.03.052 25770841

[50] Wang A Green JB Halperin JL Piccini JP Sr. Atrial fibrillation and diabetes Mellitus: jACC review topic of the week. J Am Coll Cardiol. (2019) 74:1107–15. 10.1016/j.jacc.2019.07.020 31439220

[51] Malik V Linz D Sanders P. The role of the autonomic nervous system as both “trigger and “substrate” in atrial fibrillation. Card Electrophysiol Clin. (2024) 16:271–80. 10.1016/j.ccep.2023.08.003 39084720

[52] Rafaqat S Radoman Vujacic I Gluscevic S Sharif S Klisic A. Biomarkers of diabetes: role in the pathogenesis of atrial fibrillation. Eur Rev Med Pharmacol Sci. (2024) 28:1524–40. 10.26355/eurrev_202402_35482 38436186

[53] Mohsin M Zeyad H Khalid H Gapizov A Bibi R Kamani YG The synergistic relationship between atrial fibrillation and diabetes Mellitus: implications for cardiovascular and metabolic health. Cureus. (2023) 15:e45881. 10.7759/cureus.45881 37885547 PMC10599207

[54] Băghină RM Crișan S Luca S Pătru O Lazăr MA Văcărescu C Association between inflammation and new-onset atrial fibrillation in acute coronary syndromes. J Clin Med. (2024) 13:5088. 10.3390/jcm13175088 39274304 PMC11396258

[55] Batta A Hatwal J Panda P Sharma Y Wander GS Mohan B. Impact of initial high sensitivity C-reactive protein on outcomes in nonvalvular atrial fibrillation: an observational study. Future Cardiol. (2024) 20:295–303. 10.1080/14796678.2024.2354110 39120602 PMC11318744

[56] Colak A Akinci B Diniz G Turkon H Ergonen F Yalcin H Postload hyperglycemia is associated with increased subclinical inflammation in patients with prediabetes. Scand J Clin Lab Invest. (2013) 73:422–7. 10.3109/00365513.2013.798870 23767858

[57] Daniele G Guardado Mendoza R Winnier D Fiorentino TV Pengou Z Cornell J The inflammatory status score including IL-6, TNF-alpha, osteopontin, fractalkine, MCP-1 and adiponectin underlies whole-body insulin resistance and hyperglycemia in type 2 diabetes mellitus. Acta Diabetol. (2014) 51:123–31. 10.1007/s00592-013-0543-1 24370923

[58] Esposito K Nappo F Marfella R Giugliano G Giugliano F Ciotola M Inflammatory cytokine concentrations are acutely increased by hyperglycemia in humans: role of oxidative stress. Circulation. (2002) 106:2067–72. 10.1161/01.CIR.0000034509.14906.AE 12379575

[59] Sha R Baines O Hayes A Tompkins K Kalla M Holmes AP Impact of obesity on atrial fibrillation pathogenesis and treatment options. J Am Heart Assoc. (2024) 13:e032277. 10.1161/JAHA.123.032277 38156451 PMC10863823

[60] Shu H Cheng J Li N Zhang Z Nie J Peng Y Obesity and atrial fibrillation: a narrative review from arrhythmogenic mechanisms to clinical significance. Cardiovasc Diabetol. (2023) 22:192. 10.1186/s12933-023-01913-5 37516824 PMC10387211

[61] Zhou X Dudley SC Jr. Evidence for inflammation as a driver of atrial fibrillation. Front Cardiovasc Med. (2020) 7:62. 10.3389/fcvm.2020.00062 32411723 PMC7201086

[62] Bizhanov KA Capital ACKB Baimbetov AK Sarsenbayeva AB Lyan E. Atrial fibrillation: epidemiology, pathophysiology, and clinical complications (literature review). J Cardiovasc Electrophysiol. (2023) 34:153–65. 10.1111/jce.15759 36434795

[63] Su H Ji L Xing W Zhang W Zhou H Qian X Acute hyperglycaemia enhances oxidative stress and aggravates myocardial ischaemia/reperfusion injury: role of thioredoxin-interacting protein. J Cell Mol Med. (2013) 17:181–91. 10.1111/j.1582-4934.2012.01661.x 23305039 PMC3823148

[64] Zhang ZY Pan L Dang S Wang N Zhao SY Li F Glucose fluctuations aggravate cardiomyocyte apoptosis by enhancing the interaction between txnip and akt. BMC Cardiovasc Disord. (2024) 24:470. 10.1186/s12872-024-04134-0 39223509 PMC11370038

[65] Linz D Chaldoupi SM. Longitudinal mapping of the bidirectional relationship between atrial fibrillation and cardiac remodeling. Heart Rhythm. (2024) 21:16–7. 10.1016/j.hrthm.2023.10.018 37865380

[66] Bhatti JS Sehrawat A Mishra J Sidhu IS Navik U Khullar N Oxidative stress in the pathophysiology of type 2 diabetes and related complications: current therapeutics strategies and future perspectives. Free Radic Biol Med. (2022) 184:114–34. 10.1016/j.freeradbiomed.2022.03.019 35398495

[67] Gonzalez P Lozano P Ros G Solano F. Hyperglycemia and oxidative stress: an integral, updated and critical overview of their metabolic interconnections. Int J Mol Sci. (2023) 24:9352. 10.3390/ijms24119352 37298303 PMC10253853

[68] Li F Xie M Zeng G Zhu J Zhou J Tan S Sotagliflozin attenuates atrial oxidative stress and the susceptibility to atrial fibrillation by activating the NRF2/HO-1 pathway. Int Immunopharmacol. (2025) 165:115461. 10.1016/j.intimp.2025.115461 40897116

[69] Saito S Teshima Y Fukui A Kondo H Nishio S Nakagawa M Glucose fluctuations increase the incidence of atrial fibrillation in diabetic rats. Cardiovasc Res. (2014) 104:5–14. 10.1093/cvr/cvu176 25082849

[70] Wei ZX Cai XX Fei YD Wang Q Hu XL Li C Zbtb16 increases susceptibility of atrial fibrillation in type 2 diabetic mice via txnip-Trx2 signaling. Cell Mol Life Sci. (2024) 81:88. 10.1007/s00018-024-05125-2 38349408 PMC10864461

[71] Lu S Liao Z Lu X Katschinski DM Mercola M Chen J Hyperglycemia acutely increases cytosolic reactive oxygen Species via O-linked GlcNAcylation and CaMKII activation in mouse ventricular myocytes. Circ Res. (2020) 126:e80–96. 10.1161/CIRCRESAHA.119.316288 32134364 PMC7210078

[72] Hegyi B Fasoli A Ko CY Van BW Alim CC Shen EY CaMKII serine 280 O-GlcNAcylation links diabetic hyperglycemia to proarrhythmia. Circ Res. (2021) 129:98–113. 10.1161/CIRCRESAHA.120.318402 33926209 PMC8221539

[73] Chen PS Chen LS Fishbein MC Lin SF Nattel S. Role of the autonomic nervous system in atrial fibrillation: pathophysiology and therapy. Circ Res. (2014) 114:1500–15. 10.1161/CIRCRESAHA.114.303772 24763467 PMC4043633

[74] Song Y Tan Y Deng M Shan W Zheng W Zhang B Epicardial adipose tissue, metabolic disorders, and cardiovascular diseases: recent advances classified by research methodologies. MedComm. (2023) 4:e413. 10.1002/mco2.413 37881786 PMC10594046

[75] Anderson ME Brown JH Bers DM. CaMKII in myocardial hypertrophy and heart failure. J Mol Cell Cardiol. (2011) 51:468–73. 10.1016/j.yjmcc.2011.01.012 21276796 PMC3158288

[76] Erickson JR Pereira L Wang L Han G Ferguson A Dao K Diabetic hyperglycaemia activates CaMKII and arrhythmias by O-linked glycosylation. Nature. (2013) 502:372–6. 10.1038/nature12537 24077098 PMC3801227

[77] Hegyi B Bers DM Bossuyt J. CaMKII signaling in heart diseases: emerging role in diabetic cardiomyopathy. J Mol Cell Cardiol. (2019) 127:246–59. 10.1016/j.yjmcc.2019.01.001 30633874

[78] Mesubi OO Rokita AG Abrol N Wu Y Chen B Wang Q Oxidized CaMKII and O-GlcNAcylation cause increased atrial fibrillation in diabetic mice by distinct mechanisms. J Clin Invest. (2021) 131:e95747. 10.1172/JCI95747 33151911 PMC7810480

[79] Nishio S Teshima Y Takahashi N Thuc LC Saito S Fukui A Activation of CaMKII as a key regulator of reactive oxygen species production in diabetic rat heart. J Mol Cell Cardiol. (2012) 52:1103–11. 10.1016/j.yjmcc.2012.02.006 22394624

[80] Sommese L Valverde CA Blanco P Castro MC Rueda OV Kaetzel M Ryanodine receptor phosphorylation by CaMKII promotes spontaneous ca(2+) release events in a rodent model of early stage diabetes: the arrhythmogenic substrate. Int J Cardiol. (2016) 202:394–406. 10.1016/j.ijcard.2015.09.022 26432489 PMC4872299

[81] Mustroph J Wagemann O Lucht CM Trum M Hammer KP Sag CM Empagliflozin reduces ca/calmodulin-dependent kinase II activity in isolated ventricular cardiomyocytes. ESC Heart Fail. (2018) 5:642–8. 10.1002/ehf2.12336 30117720 PMC6073019

[82] Mustroph J Baier MJ Pabel S Stehle T Trum M Provaznik Z Empagliflozin inhibits cardiac late sodium current by ca/calmodulin-dependent kinase II. Circulation. (2022) 146:1259–61. 10.1161/CIRCULATIONAHA.122.057364 36251785 PMC9586469

[83] Krammer T Baier MJ Hegner P Zschiedrich T Lukas D Wolf M Cardioprotective effects of semaglutide on isolated human ventricular myocardium. Eur J Heart Fail. (2025) 27:1315–25. 10.1002/ejhf.3644 40107718 PMC12370581

[84] Patterson E Po SS Scherlag BJ Lazzara R. Triggered firing in pulmonary veins initiated by *in vitro* autonomic nerve stimulation. Heart Rhythm. (2005) 2:624–31. 10.1016/j.hrthm.2005.02.012 15922271

[85] Yuan M Gong M Zhang Z Meng L Tse G Zhao Y Hyperglycemia induces endoplasmic Reticulum stress in atrial cardiomyocytes, and mitofusin-2 downregulation prevents mitochondrial dysfunction and subsequent cell death. Oxid Med Cell Longev. (2020) 2020:1. 10.1155/2020/6569728 PMC7603626 33149811

[86] Liu H Liu R Yang Z Xu F Li C. Effect of preinitiated glucose-insulin-potassium strategy for patients with undergoing planned percutaneous coronary intervention: a systematic review and meta-analysis. BMJ Open. (2023) 13:e073557. 10.1136/bmjopen-2023-073557 38149412 PMC10711875

[87] Udelson JE Selker HP Braunwald E. Glucose-insulin-potassium therapy for acute myocardial infarction: 50 years on and time for a relook. Circulation. (2022) 146:503–5. 10.1161/CIRCULATIONAHA.121.058740 35969651

[88] Negreiros PH Bau A Nadruz W Coelho Filho OR Matos-Souza JR Coelho OR Intensive treatment of hyperglycemia in the acute phase of myocardial infarction: the tenuous balance between effectiveness and safety - a systematic review and meta-analysis of randomized clinical trials. Rev Assoc Med Bras. (2019) 65:24–32. 10.1590/1806-9282.65.1.24 30758416

[89] Malmberg K. Prospective randomised study of intensive insulin treatment on long term survival after acute myocardial infarction in patients with diabetes mellitus. DIGAMI (diabetes mellitus, insulin glucose infusion in acute myocardial infarction) study group. Br Med J. (1997) 314:1512–5. 10.1136/bmj.314.7093.1512 9169397 PMC2126756

[90] Malmberg K Norhammar A Wedel H Rydén L. Glycometabolic state at admission: important risk marker of mortality in conventionally treated patients with diabetes mellitus and acute myocardial infarction: long-term results from the diabetes and insulin-glucose infusion in acute myocardial infarction (DIGAMI) study. Circulation. (1999) 99:2626–32. 10.1161/01.CIR.99.20.2626 10338454

[91] Preda A Montecucco F Carbone F Camici GG Lüscher TF Kraler S SGLT2 Inhibitors: from glucose-lowering to cardiovascular benefits. Cardiovasc Res. (2024) 120:443–60. 10.1093/cvr/cvae047 38456601 PMC12001887

[92] Wronka M Krzemińska J Młynarska E Rysz J Franczyk B. New insights into the use of liraglutide-impact on cardiovascular risk and microvascular outcomes. Biomedicines. (2023) 11:1159. 10.3390/biomedicines11041159 37189777 PMC10136170

[93] Mukhopadhyay P Sanyal D Chatterjee P Pandit K Ghosh S. SGLT2 inhibitors: effect on myocardial infarction and stroke in type 2 diabetes. J Clin Endocrinol Metab. (2023) 108:2134–40. 10.1210/clinem/dgad113 36856812

[94] Li N Chelu MG Birnbaum Y. Dapagliflozin for atrial fibrillation. Cardiovasc Drugs Ther. (2024) 38:1–3. 10.1007/s10557-024-07543-7 38319469 PMC11428186

[95] Apperloo EM Neuen BL Fletcher RA Jongs N Anker SD Bhatt DL Efficacy and safety of SGLT2 inhibitors with and without glucagon-like peptide 1 receptor agonists: a SMART-C collaborative meta-analysis of randomised controlled trials. Lancet Diabetes Endocrinol. (2024) 12:545–57. 10.1016/S2213-8587(24)00155-4 38991584

[96] Li W Wang Y Zhong G. Glycemic variability and the risk of atrial fibrillation: a meta-analysis. Front Endocrinol (Lausanne). (2023) 14:1126581. 10.3389/fendo.2023.1126581 37274320 PMC10232736

[97] Zelniker TA Bonaca MP Furtado RHM Mosenzon O Kuder JF Murphy SA Effect of dapagliflozin on atrial fibrillation in patients with type 2 diabetes Mellitus: insights from the DECLARE-TIMI 58 trial. Circulation. (2020) 141:1227–34. 10.1161/CIRCULATIONAHA.119.044183 31983236

[98] Lee H Park SE Kim EY. Glycemic variability impacted by SGLT2 inhibitors and GLP 1 agonists in patients with diabetes Mellitus: a systematic review and meta-analysis. J Clin Med. (2021) 10:4078. 10.3390/jcm10184078 34575189 PMC8470178

[99] Shao Q Meng L Lee S Tse G Gong M Zhang Z Empagliflozin, a sodium glucose co-transporter-2 inhibitor, alleviates atrial remodeling and improves mitochondrial function in high-fat diet/streptozotocin-induced diabetic rats. Cardiovasc Diabetol. (2019) 18:165. 10.1186/s12933-019-0964-4 31779619 PMC6882319

[100] Tudoran C Tudoran M Giurgi-Oncu C Abu-Awwad A Abu-Awwad SA Voiţă-Mekereş F. Associations between oral glucose-lowering agents and increased risk for life-threatening arrhythmias in patients with type 2 diabetes mellitus-a literature review. Medicina (B Aires). (2023) 59:1760. 10.3390/medicina59101760 PMC10608201 37893478

